# The Operative Management of Capitellum Fractures

**DOI:** 10.7759/cureus.59326

**Published:** 2024-04-29

**Authors:** Malik Majeed, Azeem Thahir, Matjia Krkovic

**Affiliations:** 1 School of Clinical Medicine, University of Cambridge, Cambridge, GBR; 2 Department of Trauma and Orthopaedics, Addenbrooke’s Hospital, Cambridge University Hospitals NHS Foundation Trust, Cambridge, GBR

**Keywords:** open reduction and internal fixation, operative management, trauma, orthopaedics, capitellum fracture

## Abstract

Capitellum fractures represent fewer than 1% of elbow fractures. Most commonly, these fractures occur secondary to either axial loading with the elbow fully extended or direct impact to the lateral aspect of the elbow. Numerous classification systems exist, with many types and subtypes. Since capitellum fractures are uncommon and fracture type varies widely, there is a lack of consensus with regard to treatment recommendations. We present a case series of seven patients with capitellum fractures, who presented between January 2016 and August 2020 to Addenbrooke’s Hospital (Cambridge, the United Kingdom). All patients were female, with an average age of 33 years. In each case, the affected elbow joint was immobilised using a backslab before open reduction and internal fixation (ORIF) was performed. Joint mobility was recorded both on the day of the injury and at clinic review postoperatively (first at two weeks and then at between four and eight weeks). The Oxford Elbow Score (OES) was measured retrospectively, relating to (1) before the injury and (2) six months after fracture reduction. ORIF was associated with a near-full return of pronation and supination by eight weeks, with flexion-extension also improving significantly. The Oxford Elbow Score at six months reached 82.0% of pre-injury scores. Overall, our results suggest that ORIF is a well-tolerated and effective treatment strategy for capitellum fractures. Future studies with a greater sample size are required to assess the outcomes across a longer period, to determine whether outcomes are maintained and continue to improve.

## Introduction

The capitellum is the lateral part of the humeral condyle that articulates with the radial head. Fractures of the capitellum are uncommon, accounting for fewer than 1% of elbow fractures [1]. Most commonly, these fractures occur secondary to either axial loading with the elbow fully extended or direct impact to the lateral aspect of the elbow. Clinically, patients most often present following a fall onto either an elbow or an outstretched hand [2]. The Bryan and Morrey classification describes four types of capitellum fracture: Type I (Hahn-Steinthal) describes a large capitellum fragment, with little or no trochlea involvement; type II (Kocher-Lorenz) involves a shear fracture of the articular cartilage, leaving a thin layer of subchondral bone; type III (Broberg-Morrey) fractures are severely comminuted; and type IV (McKee modification) fractures describe coronal shearing of both the capitellum and trochlea [3].

Since capitellum fractures are uncommon and fracture type varies widely, there is a lack of consensus with regard to treatment recommendations. Numerous approaches have been used with varying success; these include closed reduction, excision, open reduction, arthroscopy and prostheses [4]. While closed reduction and immobilisation is a popular nonoperative approach, the prolonged immobilisation (minimum of three weeks [5]) required to maintain the reduction of the capitellum can precipitate post-traumatic stiffness. This can lead to a reduced range of motion, particularly in extension. Also, failure of reduction, malunion and instability are frequently reported [6,7]. Closed reduction often results in some loss of joint mobility, mainly in extension [8]. Contrastingly, operative reduction is associated with not only a higher rate of union but also a higher rate of surgical complications, including infection and avascular necrosis [9,10]. Further research assessing joint mobility outcomes is lacking in the literature and therefore needs further investigation.

We present seven patients who attended Addenbrooke’s Hospital (Cambridge, the United Kingdom) between January 2016 and August 2020 and who were found to have sustained a capitellum fracture. Fractures were diagnosed by plain anteroposterior and lateral radiographs of the elbow and classified according to the Bryan and Morrey classification. The main outcomes assessed were (1) the range of motion and (2) the Oxford Elbow Score (OES), assessed before and after open reduction and internal fixation (ORIF). With this study, we aim to explore patient outcomes after the ORIF of a displaced capitellum. We hope that these findings can help inform future practice in treating this relatively uncommon elbow fracture.

## Materials and methods

Following the presentation to the hospital, plain radiographs were taken to check for fractures. Once confirmed, patients were initially managed with the backslab immobilisation of the elbow joint. Open reduction and internal fixation refers to surgical exploration, followed by articular fixation with compression screws (two Mini Acutrak® or Micro Acutrak® headless compression screws) and the repair of associated bone and ligament injuries. In each case, intraoperative fluoroscopy was used to confirm reduction. After surgical reduction, a collar and cuff were applied, with the elbow kept at 90° of flexion. To avoid stiffness, patients were advised to gradually begin mobilising two weeks post operation. Specifically, patients were advised to perform full pronation and supination while increasing elbow extension by approximately 20° every two weeks starting at 110° after reduction. An elbow in full extension was taken to be at 0°, with the flexion-extension range of motion measured as the range of greatest extension to the greatest flexion. Pronation and supination values were measured relative to a reference point with the arm midway between full pronation and full supination, with a maximum attainable ROM of 0°-90° for each. Rehabilitation exercises were supervised by a physiotherapist, and patients were advised to continue these exercises at home.

Patients were followed up in the orthopaedics clinic to assess the injury site and range of motion of the elbow joint, and radiographs were taken to confirm fixation. Additionally, Oxford Elbow Scores were recorded retrospectively, before the injury and six months after reduction. The Oxford Elbow Score is a 12-item patient-recorded outcome measure tool, with three domains: elbow pain, elbow function and psycho-social effects. Each item is scored from 0 to 4, totalling 0-48 across the 16 items. A score of 48 indicates a ‘normal’ elbow score. Each of the 16 items has a relative weight with respect to pain, elbow function and psycho-social effects [11]. For example, the item ‘pain in bed at night’ has a ‘loading’ of 0.88, 0.18 and 0.20 for these domains, respectively. When calculating mean domain scores as a percentage, the relative ‘loading’ of each of the 16 items with respect to each of the three domains was multiplied by the mean raw score (out of 4) for that item. For example, the mean of the eight patient scores for this item was calculated (3.1) and then multiplied by each of the three ‘loading’ values. This was repeated for each of the 16 items, and a percentage score was calculated, comparing the mean patient domain scores with the maximum possible.

## Results

All patients were female, with ages ranging from 17 to 47 years old, with an average of 33 years (Table 1). All patients presented following a fall onto an elbow or outstretched hands. As per the Bryan and Morrey classification, the most common fracture type was I (71.4%), followed by II (14.7%) and IV (14.7%). These results are summarised in Table 1.

**Table 1:** Patient demographics, injury mechanism, fracture classification and treatment. M, male; F, female; ORIF, open reduction and internal fixation

Patient	Age (years)	Gender (M/F)	Mechanism of injury	Bryan and Morrey classification	Treatment
1	47	F	Fall from bicycle and onto the elbow	IV	ORIF
2	27	F	Fall from bicycle and onto the elbow	IV	ORIF
3	37	F	Fall onto the outstretched hands	II	ORIF
4	21	F	Fall from bicycle onto the elbow	I	ORIF
5	17	F	Fall from bicycle and onto the elbow	I	ORIF
6	26	F	Fall while skiing and onto the elbow	I	ORIF
7	45	F	Fall onto the elbow	I	ORIF

Joint mobility and the Oxford Elbow Score outcome measures are outlined in Table 2. The mean domain-specific Oxford Elbow Scores (OES) are given in Table 3.

**Table 2:** The range of motion (ROM) at clinic review (two weeks and four to eight weeks) and the Oxford Elbow Score before the injury and at six months post reduction. ‘–’ indicates that follow-up was not performed (due to patient cancellation or other scheduling difficulties). ‘*’ indicates that follow-up was not continued to four to eight weeks, since postoperative recovery was sufficient for early discharge from the clinic. ORIF: open reduction and internal fixation

Patient	Treatment	Flexion-extension (°)		Pronation-supination (°)		Oxford Elbow Score	
		Two weeks	Four to eight weeks	Two weeks	Four to eight weeks	Before injury	Six months post reduction
1	ORIF	-	15-90	-	0-90	48	46
2	ORIF	40-80	10-130	0-75	0-90	48	48
3	ORIF	30-80	45-130	0-90	0-90	48	21
4	ORIF	0-130	*	0-90	*	48	44
5	ORIF	30-90	10-100	0-90	0-90	48	36
6	ORIF	-	-	-	-	48	33
7	ORIF	45-90	15-120	0-50; 0-10	0-90; 0-80	48	48
Mean		39-94	26-114	0-79; 0-71	0-90; 0-88	48	39.4

**Table 3:** Comparison of the Oxford Elbow Score (OES) domain-specific scores before injury and six months after capitellum fracture reduction (n = 7). The calculation of domain-specific scores is outlined in the Materials & Methods section of this paper.

OES domain	Mean score before injury (%)	Mean score six months after reduction (%)
Function	100	84.2
Pain	100	83.9
Psycho-social	100	78.6

Case 1

**Figure 1:**
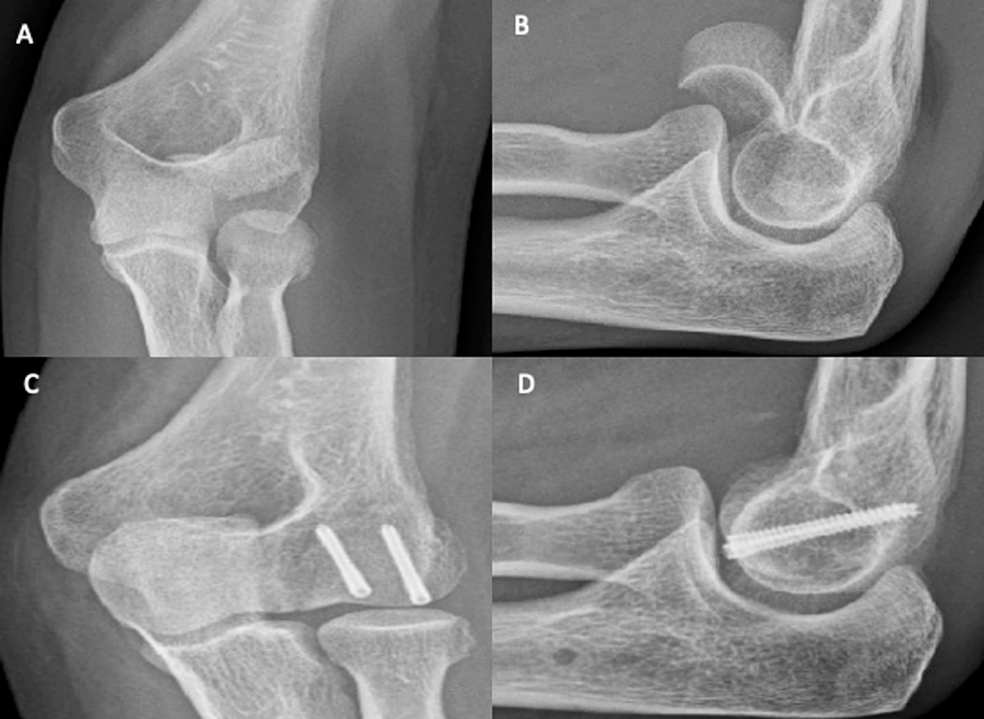
Plain elbow X-rays of case 1. Figure 1A, 1B shows anteroposterior and lateral views, respectively, of a type IV capitellum fracture. There is a large capitellum fragment, with the involvement of the lateral half of the trochlea. Figure 1C, 1D shows anteroposterior and lateral views, respectively, of the elbow after ORIF. The fracture has been successfully reduced using two Mini Acutrak® screws. ORIF: open reduction and internal fixation

A 47-year-old female presented to the emergency department after falling off her bicycle and landing on her left elbow. There was supra-olecranon swelling, with tenderness over the medial epicondyle. The patient held her elbow in 90° flexion, unable to move at the joint. The anteroposterior (Figure 1A) and lateral (Figure 1B) radiographs of her left elbow revealed a type I (Hahn-Steinthal) fracture of the left capitellum with posterior comminution and anterosuperior displacement of the distal fragment. Additionally, there was the elevation of the posterior fat pad, consistent with effusion. Closed reduction was attempted unsuccessfully. Subsequently, open reduction and internal fixation was successful. The capitellum was located distally and had flipped through 180°. Irreparable posterior comminuted fragments were discarded, and the distal capitellar fragment was reduced. The capitellum was fixed using two K-wires (30 mm and 28 mm) passed anteroposteriorly, in conjunction with two Micro Acutrak® screws (22 mm and 24 mm) (Figure 1C and Figure 1D). A collar and cuff were applied to hold the elbow in deep flexion. The patient was discharged the same day following examination and was encouraged to mobilise but avoid heavy lifting for six weeks. At the two-week review, the wound had healed, and mobilisation was encouraged. At the six-week review, the patient had regained full supination and pronation (0°-90°), with extension-flexion from 15° to 90°. Rehabilitation exercises were advised until the next review two months later.

Case 2

**Figure 2:**
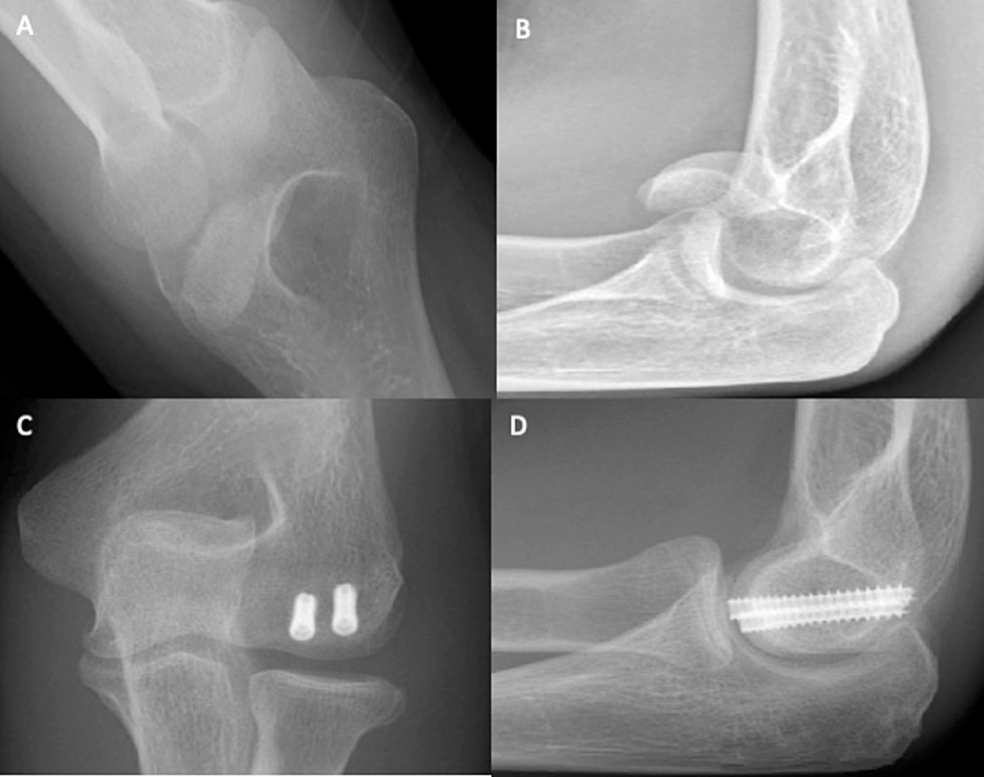
Plain elbow X-rays of case 2. Figure 2A, 2B shows anteroposterior and lateral views, respectively, of a type IV capitellum fracture. Figure 2C, 2D shows anteroposterior and lateral views, respectively, of the elbow after ORIF. ORIF: open reduction and internal fixation

**Figure 3:**
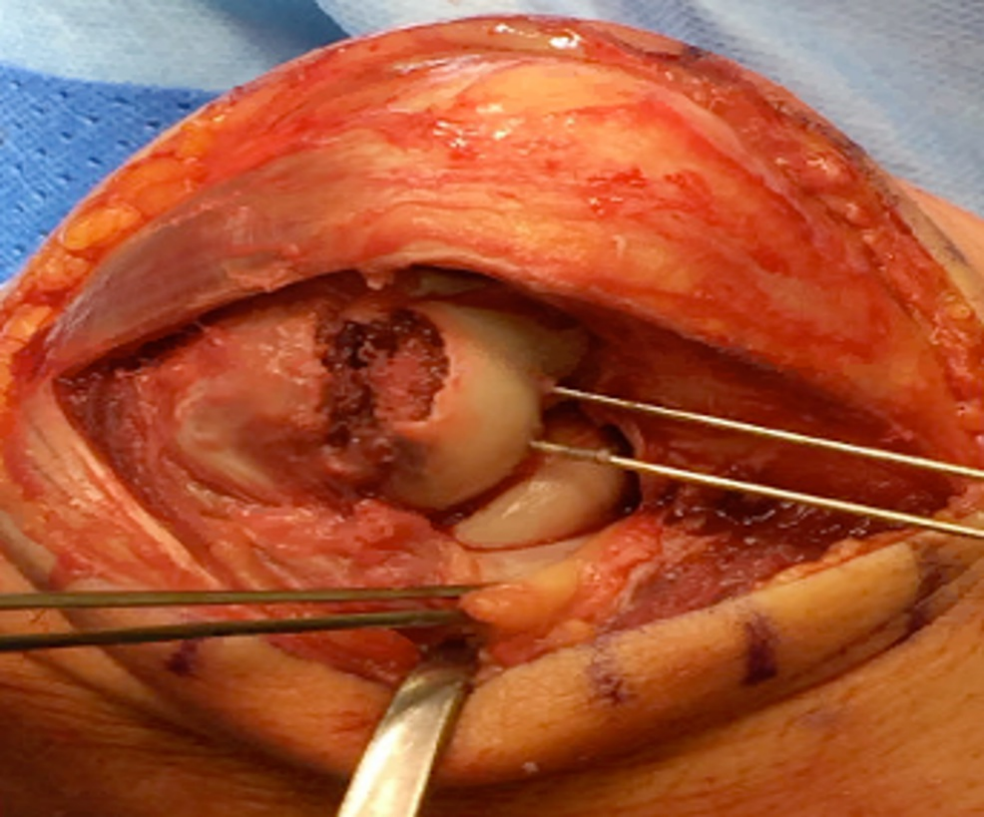
Posterior incisional approach, viewing a closed, distal humeral intra-articular fracture (case 2). The capitellum fracture extends into the trochlea, with a loss of infra-capitellum cartilage.

A 27-year-old female administrator fell off her bicycle and onto her left elbow after swerving to avoid another cyclist. The elbow was swollen, with bony tenderness along the distal humerus and radial head. The patient had been unable to move her arm following the injury. A plain radiograph of the left radius and ulna confirmed a left type IV capitellar fracture with radiocapitellar dislocation (Figure 2A and Figure 2B). Under general anaesthetic, Kocher’s manoeuvre was performed to reduce the capitellum. Despite the reduction, elbow flexion and extension were limited, with open reduction and internal fixation scheduled four days later. Open surgery revealed a closed, distal humeral intra-articular capitellum fracture, which extended into the trochlear. Also, there was reduced infra-capitellum cartilage (Figure 3). The fracture was reduced and fixed with two Mini Acutrak® screws (Figure 2C and Figure 2D). Two weeks after the surgery and beginning home rehabilitation exercises, the swelling had reduced, and the site was neurovascularly intact. Regarding the range of motion, flexion-extension was 40°-80°, with both pronation and supination at 0°-75°. At the four-month review, flexion-extension had improved to 10°-120°, and pronation and supination were now normal (0°-90°).

Case 3

**Figure 4:**
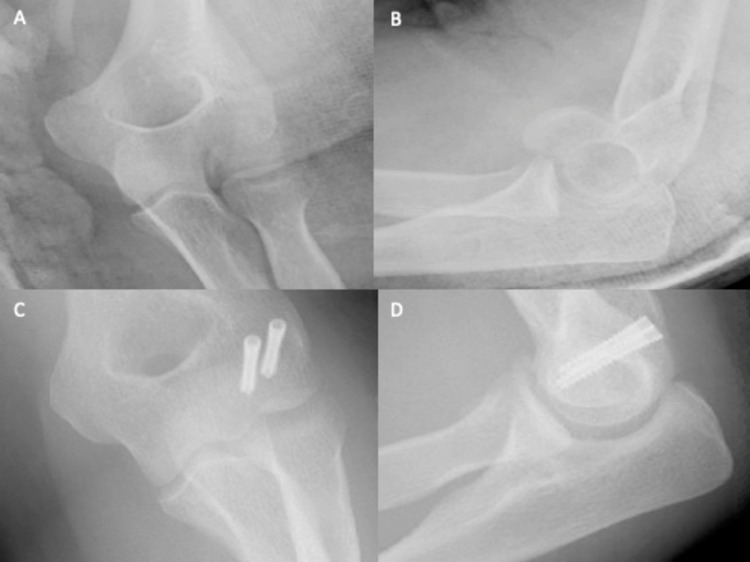
Plain elbow X-rays of case 3. Figure 4A, 4B shows anteroposterior and lateral views, respectively, of a type II capitellum fracture. Figure 4C, 4D shows anteroposterior and lateral views, respectively, of the elbow after ORIF. ORIF: open reduction and internal fixation

A 37-year-old female community nurse was walking in the countryside when she fell onto both outstretched hands. Immediately, she experienced excruciating pain, which caused her to lose consciousness briefly. In the hospital, the patient reported tenderness around both wrists and elbows. Subsequent elbow X-rays showed a type II (Kocher-Lorenz) fracture of the left capitellum (Figure 4A and Figure 4B). Four days later, ORIF was successfully performed, using 20 mm and 22 mm Mini Acutrak® two bone screws (Figure 4C and Figure 4D). At the two-week review, the wound site showed good skin apposition with no signs of infection, and elbow flexion-extension was 30°-80°, with full supination and pronation. At six weeks, flexion-extension reached 45°-130°, with extension still lacking.

Case 4

**Figure 5:**
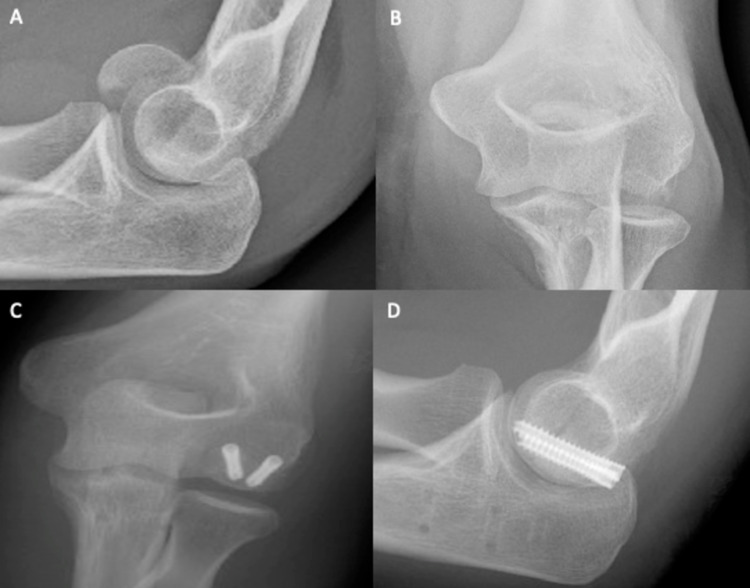
Plain elbow X-rays of case 4. Figure 5A, 5B shows anteroposterior and lateral views, respectively, of a type I capitellum fracture. Figure 5C, 5D shows anteroposterior and lateral views, respectively, of the elbow after ORIF. ORIF: open reduction and internal fixation

**Figure 6:**
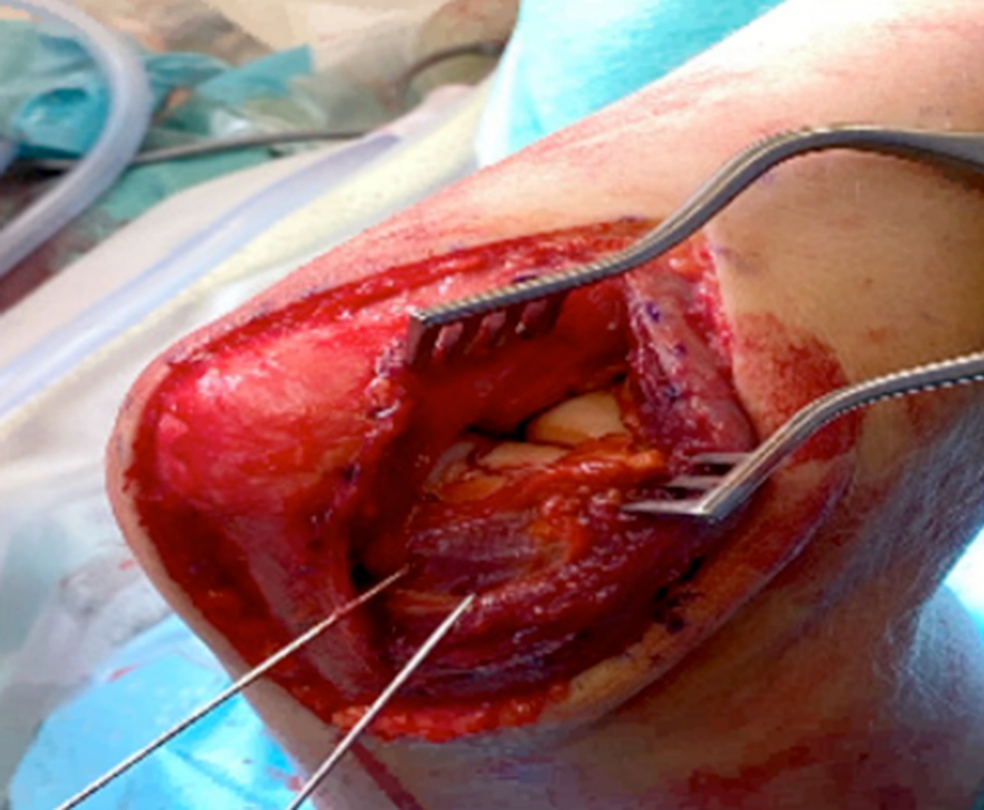
Posterior incisional approach, viewing a closed, displaced capitellar fracture (case 4).

While cycling, a 21-year-old female fell and landed on her left elbow, and her face hit the ground. She suffered a laceration to her chin and lower lip and was unable to move her elbow in the emergency department. A plain radiograph revealed a closed, displaced capitellar fracture (Figure 5A and Figure 5B). One week later, this was successfully reduced by ORIF (Figure 6) using two Mini Acutrak® screws (Figure 5C and Figure 5D). At the two-week review, the scar had healed well with no signs of infection, and the range of motion had been fully restored in all elbow movements. As such, the patient was discharged with a patient-initiated follow-up of one year, which was not needed.

Case 5

**Figure 7:**
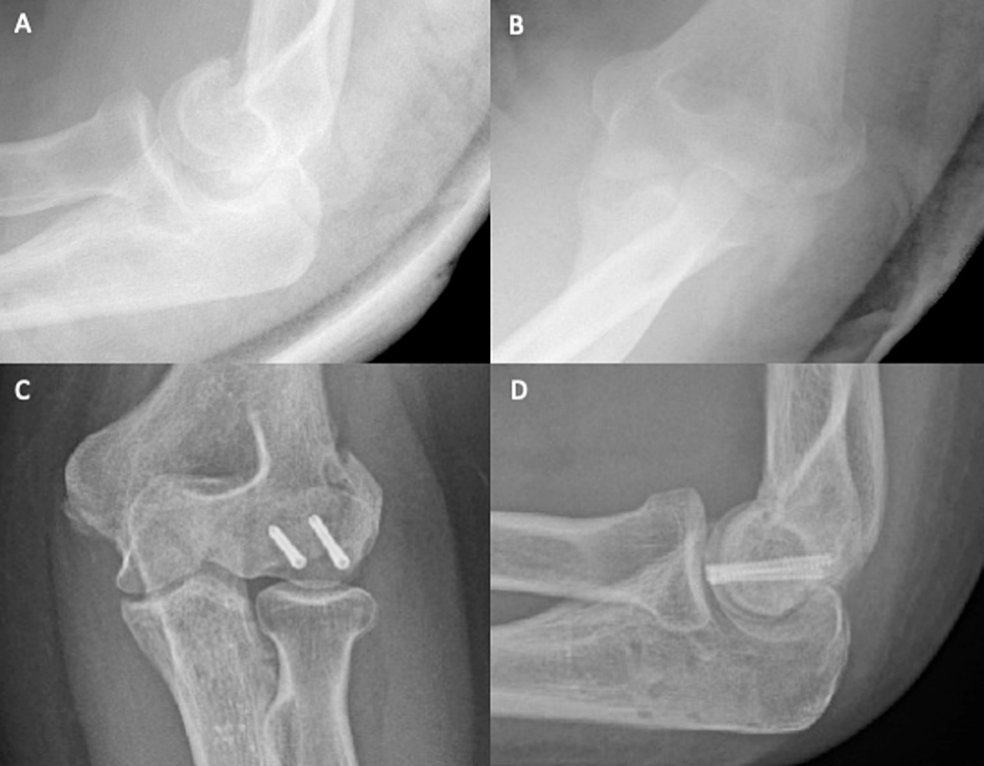
Plain elbow X-rays of case 5. Figure 7A, 7B shows anteroposterior and lateral views, respectively, of a type I capitellum fracture. The backslab applied in the emergency department can be seen. Figure 7C, 7D shows anteroposterior and lateral views, respectively, of the elbow after ORIF. ORIF: open reduction and internal fixation

A 17-year-old female care worker was reviewed in the clinic after falling off her bike and onto her left elbow 10 days previously. A plain X-ray revealed a displaced left capitellar fracture with extension into the humeral trochlea, consistent with a type IV capitellar fracture, while the lateral condyle was not displaced (Figure 7A and Figure 7B). Initially, a backslab was used to immobilise the elbow before surgery. ORIF was successful. The patient was advised to perform physiotherapy exercises, with flexion and extension advised as comfort allowed. She was advised to lift ‘nothing heavier than a cup of tea’ for six weeks. At the two-week review, flexion-extension was 30°-90°, with full pronation and supination and no tenderness. A referral was made to physiotherapy. At six weeks, flexion-extension had improved to 10°-100°, and radiographic imaging confirmed that reduction had been maintained (Figure 7C and Figure 7D).

Case 6

**Figure 8:**
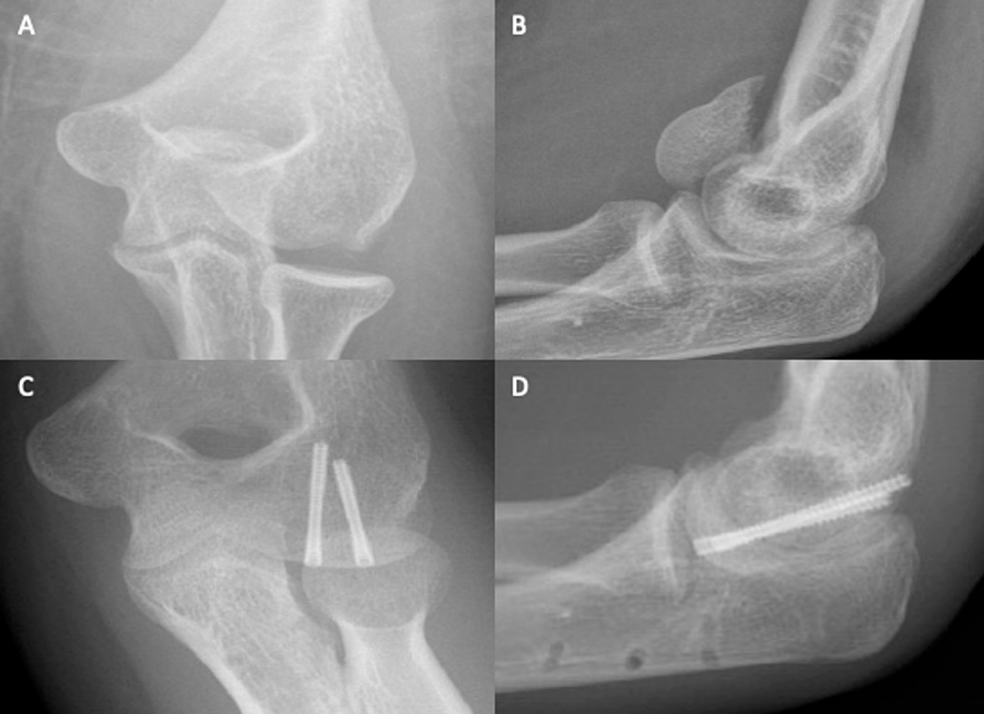
Plain elbow X-rays of case 6. Figure 8A, 8B shows anteroposterior and lateral views, respectively, of a type I capitellum fracture. Figure 8C, 8D shows anteroposterior and lateral views, respectively, of the elbow after ORIF. ORIF: open reduction and internal fixation

A 26-year-old female presented the day after falling onto her left elbow while skiing abroad. A backslab was applied to immobilise the elbow. An X-ray confirmed a moderately displaced fracture of the left lateral epicondyle (Figure 8A and Figure 8B), with associated soft tissue swelling and joint effusion. Additionally, there was a small fracture within the radiohumeral joint. The surgery involved ORIF of the capitellum using two Mini Acutrak® screws (Figure 8A and Figure 8D) and repair of the annular ligament. The latter involved the removal of the ligament from the supinator crest, with repair through interosseous drill holes. At the two-week telephone review, the patient reported difficulty with flexion and severe difficulty with extension movements. Therefore, she was advised to perform assisted active flexion and gravity-assisted extension. Three months post surgery, the patient had a full range of motion in flexion, pronation and supination but was lacking 30° of extension.

Case 7

**Figure 9:**
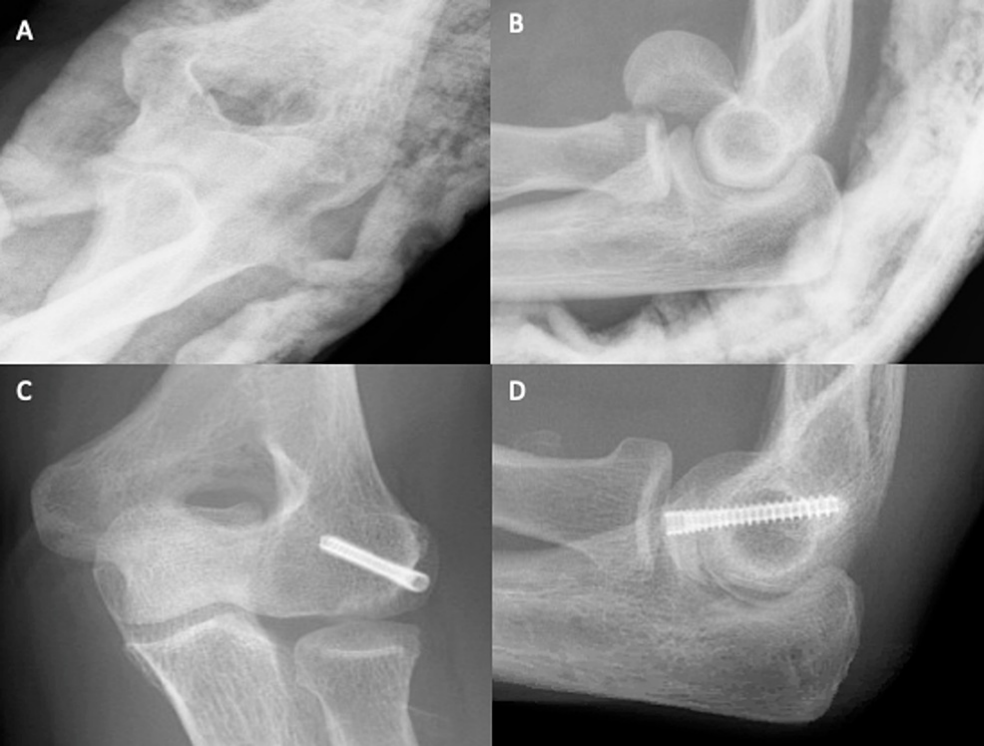
Plain elbow X-rays of case 7. Figure 9A, 9B shows anteroposterior and lateral views, respectively, of a type I capitellum fracture. The backslab applied in the emergency department can be seen. Figure 9C, 9D shows anteroposterior and lateral views, respectively, of the elbow after ORIF. ORIF: open reduction and internal fixation

A 45-year-old female caught the heel of her shoe in a grating and fell onto her left elbow. The left upper limb was neurovascularly intact, with a full range of motion of the wrist and fingers but limited elbow movement. A backslab was applied. X-ray confirmed a displaced fracture of the left capitellum, with no extension into the trochlea (Figure 9A and Figure 9B). Open surgery showed that the capitellum was dorsally displaced and rotated through 90°, with proximal migration of the radial head and the dislocation of the radiocapitellar articulation. In addition to the main capitellar fragment, several smaller fragments were seen. The capitellum was reduced by ORIF, and annular ligament repair involved two transosseous drill holes. Two weeks later, in the clinic, flexion-extension was 45°-90°, and pronation and supination were 0°-50° and 0°-10°, respectively. By six weeks, flexion-extension had improved to 15°-120°, with pronation and supination at 0°-90° and 0°-80°, respectively. An X-ray confirmed the congruency of both ulna-humeral and radiocapitellar joints (Figure 9C and Figure 9D).

## Discussion

In our study, all patients presented with symptomatic capitellum fractures after falling and landing directly either onto an elbow or onto an outstretched hand. In each case, the elbow was immobilised using an elbow backslab before proceeding with open reduction and internal fixation.

All of our patients are female, and type I (Hahn-Steinthal) fractures were most common: both of these findings are consistent with the literature [12]. Furthermore, following successful reduction, anatomical reduction was maintained in all patients throughout follow-up. This high rate of maintenance of reduction reaffirms the literature [5,12,13]. Importantly, none of our patients experienced any significant side effects, including those sometimes associated with ORIF, such as avascular necrosis and infection [9,10].

At the two-week review, the mean flexion-extension range of motion was 39°-94°, improving to 26°-114° at four to eight weeks (Table 2). Similarly, pronation and supination improved from 0-79° and 0-71° to 0-90° and 0-88°. These results demonstrate large improvements in elbow joint mobility within eight weeks after surgical reduction. Then, pronation and supination range of motion improved more quickly than did flexion-extension, which is consistent with work by Ma et al. [12]. A systematic review of 22 studies concerning ORIF of capitellum fractures found that age is not a reliable predictor of recovery of flexion-extension range of motion [5]. Therefore, while the patients in this study were 47 years old or younger, we would expect similar results in older patients. 

Regarding retrospective Oxford Elbow Scores, all patients scored 48 prior to their injury (Table 2). Six months post treatment, scores ranged from 21 to 48, with a mean of 39.4 ± 9.3. Research by Guyver et al. found that the mean Oxford Elbow Score in an asymptomatic population is 48 for each decade age group, from 11-20 to 71-80 [14]. Therefore, the patients in our study were consistent with an asymptomatic population before injury. Our post-reduction results are comparable with those following the arthroscopic fixation of the capitellum in patients with osteochondritis dissecans, where the mean OES four months postoperatively was 42 ± 4 [15]. The Oxford Elbow Score encompasses three domains: elbow pain, elbow function and psycho-social effects. Postoperative scores within each domain were 84.2%, 83.9% and 78.6% with respect to ‘function’, ‘pain’ and ‘psycho-social’, respectively (Table 3). Therefore, ‘function’ recovered the most, while ‘psycho-social’ problems were most problematic at six months. We hope that these OES findings can serve as a strong reference point for future studies. Future studies are needed to compare how overall OES and domain-specific scores progress beyond six months post reduction.

Overall, our results suggest that capitellum reduction techniques can produce strong recovery in the range of motion at the elbow at four to eight weeks of follow-up, with the mean mobility at 15°-120° in flexion-extension and 0°-90° and 0°-80° for pronation and supination, respectively. Also, fracture reduction was associated with a mean improvement of 82.0% in ‘function’, ‘pain’ and ‘psycho-social’ patient-reported outcome measures at six months.

It is important to acknowledge that our study has limitations. Firstly, the sample size is small (n = 7). This owes to the relative rarity of capitellum fractures presenting to our emergency department. Secondly, pre-injury Oxford Elbow Scores were assessed retrospectively, after fracture reduction. Therefore, these scores may not accurately reflect elbow joint mobility before the injury. However, we would expect patients to notice any significant difference between pre-injury and post-recovery joint function, which should be reflected in the difference between the OES. Additionally, while the initial clinic follow-up was within two weeks, the second review often varied between four and eight weeks, which may have affected the recorded joint mobility outcomes. Furthermore, some patients did not receive both clinic reviews at two and four to eight weeks. However, clinic review necessarily was scheduled according to both patient and doctor availability.

## Conclusions

Our study shows that ORIF is a well-tolerated and effective treatment strategy for displaced capitellum fractures. Within eight weeks, mean pronation and supination range of motion was near normal, and flexion-extension was much improved over the two clinic reviews. Joint function, pain and associated psycho-social impacts were improved by a mean of 82.0% at six months, and anatomical reduction was maintained in all patients. Future studies with a greater sample size are required to assess the outcomes across a longer period, to determine whether outcomes are maintained and continue to improve.
